# Parental perceptions of the food environment and their influence on food decisions among low-income families: a rapid review of qualitative evidence

**DOI:** 10.1186/s12889-021-12414-z

**Published:** 2022-01-05

**Authors:** Divya Ravikumar, Eleni Spyreli, Jayne Woodside, Michelle McKinley, Colette Kelly

**Affiliations:** 1 Health Promotion Research Centre, National University of Ireland Galway, University Rd, Galway, Republic of Ireland; 2 Centre for Public Health, Queen’s University Belfast, Belfast, BT12 6BJ UK

## Abstract

**Background:**

The food environment within and surrounding homes influences family dietary habits with socio-economic areas at a nutritional disadvantage. Families’ perception of the food environment and how it influences their food decisions is less clear. This rapid review aimed to synthesise qualitative evidence of parental perspectives of the food environment and their influence on food decisions among disadvantaged families.

**Method:**

Qualitative and mixed-methods peer-reviewed journal articles published after 2000, that explored the perspectives of low-income parents in relation to their food environment and how this impacted food decisions for families with children aged 2-17 years, were included in this review. Embase, Scopus and PsycINFO were the databases chosen for this review. Search strategies included seven concepts related to family, food, perceptions, influences, environment, socio-economic status and study type. Two independent reviewers screened sixty-four studies. Thematic synthesis was employed.

**Results:**

Two thousand one hundred and forty five results were identified through database searching and 1,650 were screened. Fourteen articles that originated from the US, Australia and the UK were included in this review. No articles were excluded following quality appraisal. Child preferences, financial and time constraints, and location and access to food outlets were barriers to accessing healthy food. Parental nutrition education and feeding approaches varied but positive outcomes from interventions to address these behaviours will be short-lived if inequities in health caused by poverty and access to affordable and healthy food are not addressed. The reliance on social support from families or government sources played an important role for families but are likely to be short-term solutions to health and nutritional inequities.

**Conclusions:**

This qualitative evidence synthesis provides an insight into the perceptions of low-income parents on the factors influencing food decisions. Findings have implications for public health and the development of effective strategies to improve the dietary habits of children of disadvantaged families. Sustainable changes to dietary habits for families on low-income requires policy responses to low income, food access and to the high cost of healthy foods.

## Introduction

Childhood is an important time for establishing dietary practices. The importance of the family food environment in establishing healthy eating habits during childhood and adolescence is well established [1–9]. Home food availability and parental modelling of dietary behaviours, are determinants of childhood eating behaviours. Indicatively, in three cross-sectional studies from the US, Europe and Australia; school-aged children were more likely to consume sugar-sweetened drinks when their parents reported purchasing and consuming them frequently [1–3]. Similarly, children’s preference for and intake of fruit and vegetables were predicted by home availability [4] and parental consumption of fruit and vegetables [5]. Evidence also shows that children’s eating experiences are determined by the location in which meals are consumed and whether they are consumed with other family members. In a sample of US families, meals were frequently eaten in front of the TV and this frequency was associated with lower fruit and vegetable intake and higher fat consumption amongst children [6]. Additionally, survey data highlight that family meals (even with the TV on) predicted a more balanced diet (i.e. higher consumption of vegetables, calcium-rich food, and whole grains) than not eating regular family meals [7]. Exposure to different foods is also key, as familiarity with food is a strong predictor of food preferences. Food neophobia (reluctance to eat new foods) is associated with low intake of fruit and vegetables and poor dietary quality and variety suggesting that exposure to novel foods is critical during early years [8].

The community food environment, which includes the types and location of food outlets; food availability, promotions, price and placement in stores; and media and advertising in relation to food [10], has also been shown to impact on children’s dietary intake [11]. A systematic review of twenty-six cross-sectional and longitudinal studies, examining the influence of the external food environment on children’s diets, highlights that proximity to fast-food stores is inversely associated with fruit consumption among school-aged children [11]. Food marketing is also a documented barrier to parents ability to provide a healthy diet and [12, 13] and the World Health Organisation recommends reducing children’s exposure to all types of marketing of foods and beverages high in saturated fat, salt and free sugars (HFSS) [14, 15].

Socio-economic status (SES), a social determinant of health, often related to structural and systemic issues within and across countries, is positively related to dietary quality [16] and low SES is a major determinant of food insecurity [17]. In the US, low SES is disproportionately experienced by racial and ethnic minority groups [18–21] who are also more likely to reside in poor urban areas, where residents cannot buy affordable, healthy food [22–24]. The relationship between low SES and dietary quality is also evident across Europe and among children and adolescents [25], as also shown in the Growing Up in Ireland study [26, 27]. Similarly, the National Diet and Nutrition Survey in Northern Ireland concluded that among children aged 4 to 10 years, there was an increase in consumption of confectionery, chips and other fried food and a decrease in total fruit and vegetable intake with decreasing household income [28].

The available evidence shows that the food environment (within and outside the household) influences dietary intake and can contribute to poor eating habits. However, less is known about how specific aspects of the food environment influences food decisions for families on a low-income, in part because of the predominance of quantitative studies on the food environment. Studies in deprived urban communities in Scotland and the United States highlighted the reliance on cars to access the supermarket, which predicted the frequency with which residents food-shopped [29, 30]. A mixed-methods study that examined the social dynamics of food decisions showed that individuals adapted their shopping patterns based on their financial constraints, their work and family responsibilities. In addition, they chose to shop at stores frequented by people who shared their ethnic background, income and education [31, 32]. Furthermore, Cannuscio and colleagues concluded that residents of disadvantaged communities were significantly more likely to shop at supermarkets closest to home, even if those supermarkets had a lower availability of healthful foods.

Both social and physical environments determine food choice and parents are likely to meet simultaneous and competing influences when deciding on food to buy, prepare and eat [33]. It is only through gathering the perspectives of parents themselves that one can uncover the influences parents believe are most important and should be targeted for change. The majority of the work to date utilises quantitative techniques, with no available review on low-income families’ perspectives of the influence of the food environment on food decisions. Indeed, Caspi and colleagues (2012) argue that perceived measures of the food environment may be more strongly related to dietary behaviours than objective measures (such as the density of food outlets surrounding homes) and may incorporate dimensions of food access such as psychosocial aspects (e.g. culture, economic stability, access to food outlets) that are related to the participant that would not otherwise be captured by objective measures [34]. This provided the impetus to synthesise the available qualitative evidence on parental perspectives of the food environment that influence food purchasing, meal planning and preparation among socioeconomically deprived families with children. While food environments differ across countries, key characteristics of the food environment are common with standardized tools and benchmarking indicators developed for use in over 50 countries [35]. Moreover, a qualitative synthesis captures similar concepts across studies to bring together corroborating concepts that can go beyond the content of the original study. The secondary aim of this review is to explore whether the perspectives of low-income parents on their food environment differ by family type and child developmental stage and the consequent impact that this may have on food decisions.

## Method

### Selection criteria

This rapid review included qualitative and mixed-methods (with a strong qualitative element) peer-reviewed journal articles published after 2000 that explored the perspectives of low-income parents in relation to their home and community food environment and how this impacted food decisions for families with children aged 2-17 years. According to Featherstone et al., 2015, rapid reviews can employ the same level of rigour as systematic reviews with transparency in the reporting process paramount to conducting a rapid review. In order to work within limited resources and timeframes, rapid reviews limit search parameters and databases to expedite the research process while delivering robust results [36, 37]. Studies were restricted to those from Europe, North America and Oceania and limited to English language studies only. Studies with data from other participants who were not parents were included, provided that data was not merged and parent data could be extracted. Studies with data from mixed social classes were included, provided that data from low-income/low social class parents could be extracted. Studies that contained evaluations of interventions that were not related to the food environment were excluded.

### Literature search strategy

**Fig. 1:**
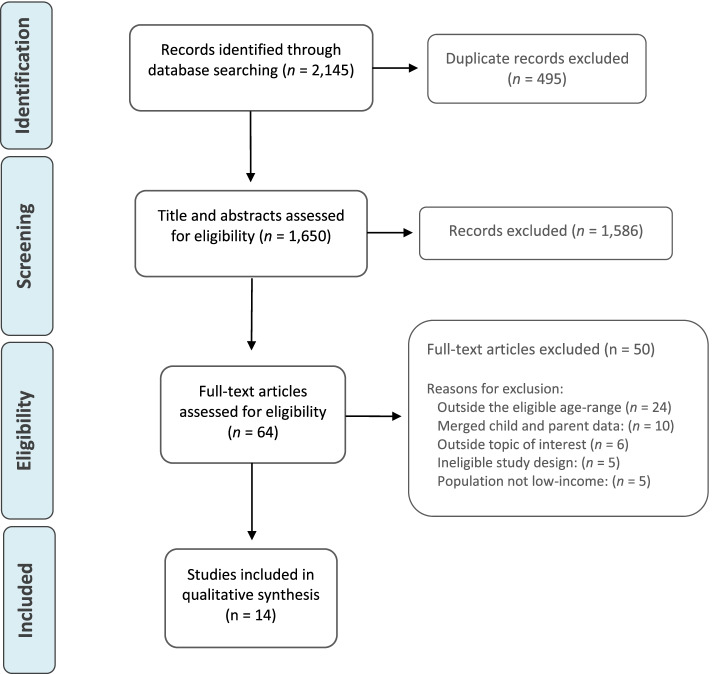
Flowchart outlining literature search results and selection process

The search strategy was developed by the team through a process of choosing search terms, searching with key terms, using truncation and wildcard searches as well as Boolean logic. An information specialist, in this case a university librarian critiqued the strategy and assisted with refining the concepts [38]. Search terms were divided into seven concepts related to family, perspectives, research design, social class, food and diet, influences and the environment. The following electronic databases were chosen for this review: Embase, Scopus and PsycINFO. These databases were chosen in order to ensure that studies examining the sociological, psychological, medical and behavioural aspects of this topic were included. In order to accommodate the limitations of the PsycINFO database, the search strategy was simplified for this search engine. The search strategy was utilised in full for both SCOPUS and Embase. The full version of the search strategy can be seen in Table 1. These searches yielded 2,145 articles. Following duplicate record removal, title and abstract screening was conducted using the selection criteria outlined above by one reviewer. Prior to screening, the title and abstract screening tool was piloted by two reviewers independently. Following this, full-text, blinded screening was conducted by two independent reviewers using Rayaan software [39]. This software blinded screening by allowing both reviewers to make decisions on each full text, without revealing the decision made by the other reviewer. When screening was complete, the results were unblinded and any conflicts could be seen by both reviewers. Conflicts were resolved by a third reviewer. A total of 14 articles were eligible for this rapid review. The inclusion and exclusion process is detailed in a PRISMA flow diagram (Fig. 1) [40].

**Table 1:** Full version of search strategy used in literature search

Search Number	Search String
#1	parent* OR famil*OR caregiver* OR mother* OR father* OR child* OR “caregiver”
#2	perspective* OR perception* OR thought* OR feeling* OR opinion* OR view OR attitude* OR beliefs
#3	qualitative OR “mixed methods”
#4	“low-income” OR “low-socioeconomic” OR deprived OR disadvantaged OR deprivation OR “low-income” OR minority OR “food poverty” OR “low socio-economic class” OR impoverished OR poor OR poverty OR “food insecurity”
#5	food* OR nutritio* OR diet* OR meal* OR snack* OR purchas* Or eat* OR prep* OR cook* OR din* OR provid*
#6	influen* OR impact OR effect* OR affect* OR factor*
#7	environment* OR ecosystem OR “urban area” OR surrounding* OR “retail environment” OR “shopping mall” OR “shopping centre” OR retail OR “grocery store” OR “food retail” OR communit* OR neighbourhood OR neighborhood OR “retail food environment” OR store* OR shop* OR “local shop” OR “convenience store” OR supermarket* OR setting* OR home* OR house*
#8	#1 AND #2 AND #3 AND #4 AND #5 AND #6 AND #7

### Quality appraisal

The Standards for Reporting Qualitative Research (SRQR) tool was used for quality assessment of included studies [41]. The SRQR consists of twenty-one items that cover aspects such as rigour, ethical issues, appropriateness of data collection methods, techniques to enhance trustworthiness, conflicts of interest and funding. Each item in the SRQR is accompanied by a descriptor [41]. This was used to determine whether the article sufficiently addressed the items covered in the descriptor, partially addressed the items in the descriptor or did not provide any information required by the descriptor. A SRQR score of 21 meant that all items were addressed sufficiently, whereas a score of 16 meant that sixteen items were sufficiently addressed and five items were either partially addressed or not addressed at all. This method was chosen due to its comprehensive nature and its ability to be implemented by novice and experienced reviewers.

### Data extraction

Data were extracted by two reviewers and stored using a standardised template on Microsoft Excel. Extracted data included theoretical background, demographic data, study aim, study setting, sampling methods, data management, analysis methods, main findings and quotations.

### Synthesis of findings

There are a number of approaches to synthesis of qualitative research, including meta-ethnography and meta-synthesis [42, 43]. Thematic synthesis, an established method for synthesis of qualitative research in systematic reviews, was employed for this review [44]. Key to this approach is the translation of concepts across studies. Data for synthesis included text labelled as ‘results’ and ‘findings’ in articles, that were extracted in the template described above and subsequently copied into NVivo 12 (QSR International Pty Ltd., Victoria, Australia). Data were then coded line by line. Following this, codes were grouped into associated related areas to construct descriptive themes. The descriptive themes were then compared to refine the relationship between them and to consequently generate analytical themes. All results and findings from the primary studies were coded. This was in order to avoid bias that may arise from using the research question as an *a priori* framework to extract data [44]. Line by line coding was conducted by two reviewers, each of whom coded half the studies. To ensure consistency in coding between them, the reviewers coded the same study at the beginning of and halfway through the coding process. They then met, discussed their codes and resolved any disagreements. Upon completion of coding, a comprehensive list of all generated codes was produced. Codes were then grouped into related topics and further condensed into themes through consensus.

## Results

### Study Characteristics

Fourteen studies were included in this rapid review exploring parental perspectives of the environmental factors that influence food decisions for low-income families within their community environment. Studies were mainly conducted in the US, two studies were from Australia and one study was from the UK. Mothers’ perspectives were explored in all studies, while eight studies also included fathers and one study included grandmothers. The majority of participants in eight out of fourteen studies were from racial or ethnic minority populations. A summary of the study characteristics can be seen in Table 2.

**Table 2:** Demographic characteristics of included studies

	Study ID, Country	Study aim	Demographics			Data collection & analysis approach	Quality
			Participants, Number, Age	Child age	Socioeconomic indicator, Ethnicity		SRQR Score
1	Agrawal 2019, US [54]	To understand how changes in low-income mothers’ work, home, and childcare environments impact their food practices for young children	Mothers (*N*=19) 23-44 years	3-4 years	"Head Start" recipients White (*N*=18), Black (*N*=1)	Individual interviews (X2) Grounded theory	13
2	Alcazar 2017, US [53]	To explore the adoption of Brighter Bites healthy eating strategies in low-income Spanish-speaking families, as well as barriers to the sustainability of improved dietary behaviors	Mothers (*N*=5)				
age not specified	School age						
	"National School Lunch Program" recipients Hispanic	Photovoice Thematic analysis	13				
3	Berge 2016, US [56]	To identify meal-level characteristics within ethnically and socio-economically diverse households that were similar and/or different between households with and without an overweight/obese child	Mothers & fathers (*N*=118) 25-65 years	6-12 years	Majority <$35,000 annually African American (*N*=77), White (*N*=15), Mixed (*N*=28)	Individual interviews Content analysis	15
4	Berge 2019, US [55]	To identify qualitative themes regarding parents’ perspectives about meal characteristics and meal types that influence family meal frequency	Mothers & fathers (*N*=150) 34.5 ± 7.1 years	5-7 years	Majority <$35,000 annually White (*N*=27), Asian (*N*=25), Somali (*N*=25), African American (*N*=22), Native American (*N*=21), Hispanic (*N*=23), Mixed (*N*=7)	Individual interviews Content analysis	17
5	Chen 2014, US [57]	To evaluate the impact of an intervention promoting ethnic produce through classroom food demonstrations, tastings and home cooking activities among ethnically diverse elementary-school children	Mothers & fathers (*N*=28) age not specified	5-8 years	Majority free and/or reduced-price school meals recipients White (*N*=14), Latino (*N*=8), Hmong (*N*=6)	Focus groups Grounded theory	16
6	Hardcastle 2016, UK [50]	To explore the perceptions and attitudes that underlie food choices, and, the impact of a school-based healthy eating intervention in mothers from an economically disadvantaged community	Mothers (*N*=16) 42.8 ± 3.5 years	13-15 years	Socially deprived area Ethnicity not specified	Individual phone interviews Thematic analysis	17
7	Harmon 2015, US [58]	To explore children’s involvement in meal preparation at home and to examine changes in children’s attitudes and self-efficacy related to cooking	Mothers, fathers & grandmothers (*N*=13) age not specified	9-12 years	Attending high-poverty and low-performing schools African American (*N*=64), Hispanic (*N*=1)	Individual phone interviews Thematic analysis	15
8	Herman 2012, US [45]	To understand the contextual factors that influence how low-income mothers felt about addressing behavioral targets for preventing obesity and their aspirations in feeding their children	Mothers (*N*=32) 20-41 years	3-5.5 years	"Supplemental Nutrition Assistance Program" recipients Black (*N*=29), οther non-White (*N*=3)	Focus groups Thematic analysis	21
9	MacNell 2017, US [46]	To show how residents of an urban food desert navigate and understand their food environments	Mothers (*N*=42) age not specified	2-8 years	"Supplemental Nutrition Assistance Program" recipients African American (*N*=31), Hispanic (*N*=10), Mixed (*N*=1)	Individual interviews Thematic analysis	8
10	Penilla 2017, US [51]	To explore parents’ experiences in providing meals and opportunities to play to their children aged 2 to 5 years	Mothers & fathers (*N*=49) 30±5.8, 35±9.1 respectively	2-5 years	Low-income area Latino	Focus groups Conceptual framework analysis	16

### Quality Appraisal

**Fig 2.**
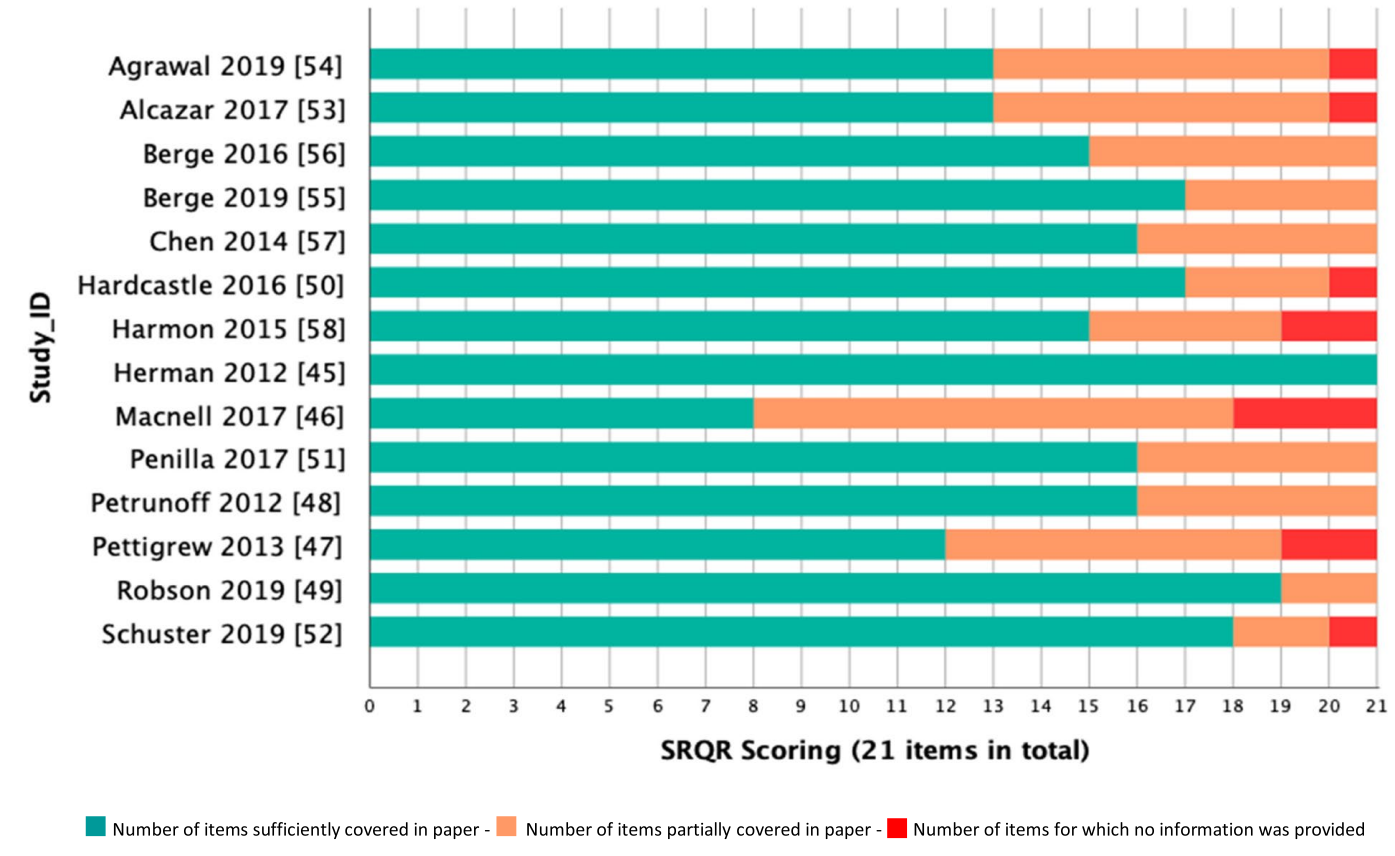
Graph outlining Standards of Reporting Qualitative Research (SRQR) scores for each included study

Thirteen of the fourteen studies provided sufficient information to meet the criteria for over half of the items outlined by the SRQR tool. One study in this review, Herman et al., 2012, provided sufficient information for all 21 items in the SRQR tool [45]. One study, Macnell et al., 2017, provided no information or partial information for the majority of the 21 items [46]. No studies were excluded on the basis of this quality assessment as they may potentially offer valuable insight. A graph outlining the findings from the quality appraisal can be seen in Fig. 2. A traffic light system has been used to indicate whether the article sufficiently addressed the items covered in the descriptor (green), partially addressed the items in the descriptor (orange) or did not provide any information required by the descriptor (red).

### Thematic Synthesis

Thematic synthesis yielded the following three themes: ‘purchasing,’ ‘planning’ and ‘preparation.’ Each theme reflects a stage of the decision-making process around food and each subtheme highlights an environmental factor that influences parents’ decision making at that specific stage. The quotations presented under each theme and subtheme were chosen for the purpose of best illustrating the theme and subtheme. Quotations were selected and discussed by two reviewers.

#### PURCHASING

##### Financial constraints

Parents frequently reported that they struggled with money, budgeting and limited income and this influenced food decisions and usually dictated the food provided for their families.

Food cost

Convenience [47, 48], quality and freshness [46], were often weighed up against food price to determine food purchases. Food cost was a primary influence of food choices for parents [46–50], with parents often opting for what is cheap or on offer [47, 50]. The high cost of food often led to unhealthy food purchases [51], with healthy food considered to be expensive [48]. Parents were also driven by sales and coupons [49]. Fresh fruit and vegetables were considered expensive when compared to frozen food and energy-dense snacks, “Coz there's like five of us…well you could get twenty penguin bars (chocolate bar) for a pound and sometimes you can't even get five apples for a pound…” [50].

Budgeting with a limited income

Parents’ limited income was attributed to poor economic circumstances which resulted in financial and time constraints [51], and difficulties securing nutritious food [52]. “It’s hard…gas to water and electricity, has gone up. Even rent…plus I have car problems and that’s just cutting my budget to buy healthy food even more” [47]. This struggle was particularly difficult for single mothers [50, 53]. Budgeting techniques by parents included “decreasing variety of foods, substitution of cheaper, lower-nutrient foods” and making strategic food purchases, “look at the cost of a whole pineapple, a frozen pineapple, canned pineapple, and save the most money without jeopardizing your nutrients” [52]. Parents also budgeted by reducing portion sizes or skipping meals in order to ensure enough food for their children [46, 52].

Food assistance

Many parents relied on food assistance to pay for all of their food or to augment their food budget. This was specific to US parents [45, 46, 52–54]. However, food assistance often ran out towards the end of the month and parents struggled to ensure enough food for their families, “but at the end if I don’t have enough on my [food assistance] card I can’t buy fruits and vegetables” [52]. Free school meals also assisted food provision for children in the UK [50]. However food in US schools and high schools was reported as being high in fat and carbohydrates [51]. Australian parents reported that they found childcare useful in providing healthy meals for their children [48]. Food provided in some US childcare settings was perceived negatively due to large quantities of food provided, “My child is two years old, weighs 40 pounds and eats five times per day because of the routine at day care, they [children] eat breakfast, a snack, lunch, a snack, an afternoon meal” [51].

##### Location and access to food outlets

A high prevalence of fast-food outlets in US communities was noted by parents [53], with one study commenting that “Every corner is a liquor store, or fast-food” [51]. Local communities were also populated with small shops or convenience stores which parents avoided due to high cost and budget constraints: “the food is too high [in price] at these small stores . . . You can’t afford to buy stuff” [46]. Instead they aimed to find the best prices by travelling further away [46] and shopping at multiple stores [46, 49, 52]. A lack of personal transport affected shopping locations and the frequency of shopping trips [46]. Public transport such as buses were considered to be “time-consuming and inconvenient” and taxis were expensive and this caused parents to shop once per month, directly after receiving food assistance [46]. This reduced the amount of perishable produce that was bought and resulted in difficulty transporting and storing food as well as planning meals for one month [46]. Having to accept lifts from friends or relatives also resulted in parents’ reduced authority over food decisions [46].

#### PLANNING

##### Child preferences

Child preferences guided food choices amongst parents. Parents often bought food based on a child’s likes or dislikes or their faddishness/fussiness, “So now, I am going to the green seedless grapes . . . this is just what my son likes,” [49]. Sometimes this was done to manage stressful mealtimes or because it was important to parents that children liked their food [54]. Family meal options were also decided based on what children or other family members asked for, “I usually ask them, you know, what they want to eat for dinner,” [55]. Parents often struggled with child pestering and this was highlighted as an influence of food choices, “I give in to her because…she just bats her eyes and she’s like, ‘Ma, please?’” [45]. This was further heightened by marketing targeted specifically at children which was a driver of food purchases, “Mom, get the car mac and cheese! Get the car mac and cheese! Will you get the car mac and cheese?” [49].

##### Parental feeding approaches

Parental food-related practices varied considerably across selected studies and participants described a number of approaches to shape their children’s eating behaviour [45–48, 50, 52, 53, 55]. Avoiding restrictions, setting limits, being responsive and offering culturally specific foods were some of the feeding approaches voiced in the literature and these influenced the planning of family meals.

Avoiding food restrictions

Some parents did not enforce food restrictions due to the belief that there would be adverse consequences to their child’s diet and relationship with food [47, 48, 52]. Applying restrictions on snacks or portion sizes was perceived to lead children developing an obsession with the restricted food, body image issues and eating disorders, “I don’t want to point it out to her because I don’t want her to have an eating disorder” [52]. Additionally, there was no perceived benefit in limiting child’s access to foods and snacks, as doing so would cause increased mealtime stress and misbehaving, something that parents were keen to prevent. Studies described parents’ effort to focus on the healthy aspects of children’s diet and encourage these further, instead of refusing them the consumption of occasional treats or closely monitoring their food intake [48, 52].

Setting limits

In contrast to the views expressed above, parents in a few studies voiced the idea that they should be setting certain limits in terms of what foods children should be allowed to eat [45, 48, 52, 55, 56]. Study participants talked about food rules that were related, but not limited to, reducing the consumption of unhealthy snacks (e.g. soft drinks) [45, 48], maintaining a frequent intake of fruit and vegetables [45], as well as and trying novel and disliked foods [41, 46, 50]. The food rules were created according to parents’ nutrition- and health-related goals and concerns, “When I’m home, you have to eat vegetables in my house” [45]. Many rules around family meals often had to do with the use of electronics. Even though some families admitted to have dinner in front of the TV, the majority of parents did not allow TV or phones while they ate, as they distract children from their food and from interacting with the rest of the family [55, 56], “I don’t want them to be distracted from eating, because after a while it gets cold and they don’t want to eat it” [55].

Being responsive to child’s cues

Most studies reported that parents refrained from using pressurising approaches when feeding their children [52, 55, 56]. Berge et al. 2016, reported parental tendencies to enforce a clean-plate rule among parents of overweight and obese children. Being responsive to the cues of children and allowing them to determine when they are full was seen as an integral part of helping them establish a healthy relationship with food and listen to their appetite sensations [45, 48, 52, 56], “I’ve always told her, if you’re full, stop eating. You don’t have to finish it, don’t stuff yourself” [56]. Additionally, responding promptly to the eating patterns of children was perceived beneficial to maintaining a positive interaction during family mealtimes. Instead of forcing child to clean their plate, parents described how they provided alternative foods which their child preferred eating or allowed the child to finish eating after trying the served food once: “You don’t have to eat everything as long as you try it. If you’re not hungry, just eat a little something” [56].

Food to modify child’s behaviours

In two studies participants admitted that the desire to avoid conflict over food and keep the child content made them adopt a more lenient approach by satisfying child’s food requests [45, 48, 52]. Bribing children with food was also mentioned in three studies as an established technique to encourage a child to adopt a certain behaviour including eating healthy foods [48, 52, 55], “I use a bribe especially for my little boy, three-year-old, if I have to go up the shops if you are a good boy you can have a lollipop” [48]. Food was also used as a reward from parents or to commemorate a child’s achievement, “You guys did a good job” [55].

Modelling healthy eating habits

Parents expressed awareness of children’s tendency to copy any dietary habits they demonstrated and explained how they used it to promote healthy eating: “When my son decides to eat a 100% natural fruit instead of junk food, [it] shows me that the healthy habits I practice in our diet transcend the decisions he makes in his nutrition” [53]. Parents were also aware of their positions as “role models” for their children in relation to healthy eating and the power that this could have [48].

Introducing culturally specific foods

Ethnicity and culture played an important role in diet of low-income families and their meals often featured traditional recipes and culturally specific foods [46, 52, 53, 55]. Parents expressed the desire to familiarise their children with their ethnic cuisine in an effort to share their cultural heritage with them, but also to introduce a wider variety of foods into their diet [52, 55], “Um, I guess we’ve been trying to teach our kids some other culture foods that we grew up with and teaching them how to gain a taste for it. Instead of just eating strictly, you know, American-style food, like sausage, corn dogs, and pizza.” [55]. Additionally, in studies that recruited parents with an immigration background, participants explained that they chose food stores based on their availability of foods from their home country [46, 52, 55], “they sell more things there from my country—things that Wal-Mart has little or none of” [46].

Parents’ own childhood experiences around food

Through closer reflection on their feeding approaches, parents highlighted that their own experiences from childhood had an impact on the feeding approaches they adopted as parents [45, 47, 50, 52, 55, 56]. Specifically, participants shared experiences of having grown up in food insecure households with poor dietary variety and limited access to fruit and vegetables, as well as sweets and snacks. Further negative childhood memories had to do with their parents’ increased use of authority, food restrictions and pressure to eat: “My mom didn’t allow that. We were at the table, we would finish what we were eating … If I was done and my food was still on the plate, I still had to sit there. I’ve fallen asleep at the table many times” [52]. The determination to create positive dietary experiences for their own children led them to follow different parenting approaches from those employed by their parents. These approaches included eating together as a family [50, 52, 55, 56], avoiding conflict over food and satisfying children’s food requests, even when these foods were considered to be unhealthy [45, 47, 52]. Moreover, participants in a study by Schuster and colleagues recounted their poor dietary practices in childhood and tried to ensure their own children had a balanced diet and diverse nutrition: “I don’t want her being like me. You know, with the junk food, with soda. I want her to grow up being healthier and not developing those bad habits like me.” [52].

##### Work and time constraints

Lack of time was frequently mentioned in the included studies when conversations addressed food preparation and family meals [47, 48, 52, 54, 55]. Busy daily schedules due to parental work and children’s school were quoted to leave limited time for cooking home-made food and planning of family meals. As a result, parents admitted to resorting to quick, convenient meals which, as described in the study findings were of lower nutritional quality (e.g. fast-food, frozen meals), [48, 52, 55]. This was a concern for parents: “I worry because I want to get them that healthy food so you know they get what they need. But sometimes I have to turn to the hotdog” [52]. Berge et al., 2019, discussed that hectic schedules did not allow family members to share meals frequently within the week, whereas at the weekend they were more flexible and therefore, could enjoy more meals within the day together [55].

#### PREPARATION

##### Social support

Nutrition interventions and cooking programs

Nutrition intervention programs offered hands-on training in preparing healthy recipes and included a nutrition education component for children from socioeconomically deprived families [50, 53, 57, 58]. Additionally, the Brighter Bites program, described by Alcazar et al., 2017, provided parents with fresh fruit and vegetables for their families. There was a long-term positive impact on both parents’ and children’s cooking skills and attitudes towards preparing healthy and balanced meals at home [53]. Recipes prepared at school offered an opportunity for children to try new fruit and vegetables and as a result, parents observed an increase in their children’s preference to and consumption of vegetables at home, [50, 58]. Ultimately, parents explained that their families modified their dietary habits by opting for more fresh fruit and vegetables and healthier cooking methods based on what they learned during the programs: “multiple parents mentioned their children developed more positive attitudes toward fruits and vegetables,” [57].

##### Sources of information on healthy food/balanced diet

Parents discussed the various sources of information in their environment that contributed to their knowledge around food and health and ultimately, influenced the planning of meals for themselves and their families.

Family and friends

Nutrition-related advice from relatives and friends was actively sought out by low-income parents and used to determine which foods would be purchased or avoided. This information was “readily understood and was assumed to have high credibility” [47].

Food labelling and TV

Lack of familiarity with the nutritional terminology on the packaging, as well as confusion around percentages of daily intakes compromised the usefulness of nutritional information and nutritional claims for low-income parents, “Like, a percentage of your daily intake, I don’t really get that. And I don’t know what the actual intake is for me” [47]. Television also influenced perceptions around food through nutrition- and health-related messaging and through raising awareness of products’ nutritional content and properties as part of marketing. On the other hand, the overflow of nutritional information from television and food packaging was often quoted as being contradictory and parents were left in a state of confusion regarding suitability and healthfulness of foods for their children, “I feel actually annoyed at that, because I think when you want to be good, what do you choose?” [47]. Advertising on TV and in stores and restaurants also impacted what children wanted to eat and thereby influenced food planning by parents [51].

Two studies reported parental concerns about additives in foods [47, 48]. The consumption of foods containing colourings and preservatives was perceived to affect children’s behaviour by causing irritability and mood changes. In this way, food shopping was influenced by the presence of food additives which was often described to be a more important guide than other aspects of foods’ nutritional value. As pointed out by the authors of one study, increased concern regarding food additives may have been a consequence of the attention given by the Australian media [47]. Avoidance of dietary fat also guided food decisions (preference for low-fat products) with the absence of other dietary considerations [47, 49].

Parents’ nutrition knowledge and related food behaviours

Low-income parents expressed the desire to provide their children with safe and nutritious food and explained that healthiness was a vital consideration when planning and preparing meals for their family [47, 51–53, 55]. As described in two studies, an important tool to implement healthy dietary habits was to increase their family’s intake of fruit and vegetables [53, 55]. The health-related benefits of a diet rich in fruit and vegetables encouraged participants to disregard their high cost in order to provide their family with fresh fruit and vegetables [53]. Including more vegetables with their main meals and offering their children fruit as a snack were methods used to achieve this goal: “I try to add in vegetables and make it as healthy as I can, like boiling vegetables with meat or adding, like, fruits on the sides” [55]. However, fruit and vegetables were also perceived to carry an unnecessary degree of risk due to the possibility that they may need to be discarded [47]. In addition, parents’ attempts to avoid unhealthy snacks were addressed [47–49, 52]. This also highlighted misconceptions about healthy and unhealthy snacks when planning which foods to buy, “I was gonna get pretzels. Kind of healthier than chips for the kids to eat. I was trying to get healthy snacks” [49]. Parents also voiced concerns regarding their children’s consumption of high-sugar foods and drinks due to the negative effect of these foods on their children’s dental health [47].

#### Influence of other adults

Aside from purchasing, planning and preparation, other adults were perceived to influence the food consumed by children. This influence came from siblings or friends [54], as well as other caregivers in schools and childcare [52]. Accepting this support, however, often made it difficult for parents to implement structure and rules for feeding children and resulted in the undermining of the parents’ authority [45]. Parents reported that other adults often provided unhealthy food to children “There’s always something sitting out and they just go over there and get it. And it’s usually not very healthy stuff” [54]. This was a particular issue with grandparents, who provided children with coffee, soda, juice [45] or ice-cream for breakfast and were generally considered an unhealthy influence [51]. Reasons for this included grandparents giving in to children’s whining and pouting [51] or wanting to keep children happy, “I’m not going to be here that much longer so I want them to love me and be happy with me” [45]. Social support also came from partners with a clear division of responsibilities being viewed positively by parents, despite this not always occurring in practice [48]. Some parents “tag-teamed” all aspects of family meals including food shopping, preparation, cooking and washing up after meals. However, food preparation and cooking were more commonly performed by mothers [55].

## Discussion

This rapid review primarily aimed to synthesise qualitative findings of parental perspectives of the food environment and their influence on food decisions among low-income families with children. The synthesis offers a novel insight into the plethora of contextual factors within and surrounding the home that parents perceive to influence their food purchasing, planning and preparation. Some of these factors were within their control such as a desire to provide a healthy diet, approaches to feeding and take-up of nutrition programmes and cooking interventions. Social determinants such as marketing and advertising, food availability and financial or time constraints were outside of their direct control. These social determinants leave parents with little control over what they can buy or prepare for their children and nutrition/healthy eating interventions at the family level likely to be ineffective without addressing the root cause of (food) poverty.

The secondary aim, to explore whether the perspectives of low-income parents differed by family type and child developmental stage, was not met. Two studies briefly addressed single parent families and reported that this increased financial constraints. However, no further references to family type were found and differences by developmental stage were also not possible to explore. Most studies captured the views of parents with young children, with only one study exploring the perspectives of parents with adolescents.

Across all studies, the number of participants from racial and ethnic minority groups indicates structural racism, health and nutritional inequity and food poverty [59]. Recent evidence also suggests that the COVID-19 pandemic has exacerbated these disparities in food security [60]. The review also highlights the systemic impact of poverty on healthy eating efforts amongst low-income families, who are predominately from minority groups.

### Providing healthy food

Parents tried to make food choices that would nourish their children and parents reported the use of food rules, such as limiting the number of unhealthy snacks and screen limits during mealtimes, which are associated with maintenance of a healthy weight among young people [61, 62]. However, perceptions around diet and health varied considerably in terms of accuracy, reflecting different levels of nutrition knowledge. This aligns with other work [63, 64] and supports the need for further education that targets low-income parents. Similarly, this review showed that parents often accommodated fussy-eating and yielded to pestering from children and this impacted food purchased and prepared at home. Evidence suggests that children whose parents often give in to repeated food requests have poorer diets and are more likely to be overweight [65].

The positive influence of cooking interventions on families’ food decisions was discussed in the included literature. Aside from the measurable benefits of these initiatives [57, 66], the qualitative data suggest that the positive experiences gained though participation in these programmes motivated families to adopt healthier cooking patterns. Additionally, children became involved in meal preparation and acquired a positive attitude towards healthier foods. Experiential learning, as utilised from these initiatives, promotes immediate processing of information [67], and has been shown to be an effective method in promoting nutritional knowledge and changing attitudes towards identifying nutritious foods, shopping and cooking amongst low-income families [68, 69].

### Food availability and accessibility

Food environments were characterised by convenience stores and a high prevalence of fast-food outlets. This supports research which indicates that low-income areas are more heavily populated with fast-food outlets [70–72], and less likely to have large supermarkets, resulting in reduced availability of fruit, vegetables and low-fat dairy products to families [10, 70, 73]. Research shows that access to healthy food outlets increases fruit and vegetable consumption in children [74], while the presence of fast-food outlets surrounding schools and homes reduces fruit and vegetable intake in this population, making the food environment key in impacting food decisions [75, 76]. In parallel, a scarcity of large supermarkets in local areas caused parents to travel outside of their neighbourhoods to larger supermarkets and a lack of personal transport caused parents to rely on stock-piling and non-perishable goods. Research confirms that stockpiling increases amount and frequency of convenience product consumption [77, 78], while greater availability of perishable foods such as fresh produce is associated with increased consumption. This makes availability of fruit and vegetables a key target for change [79]. Health promotion and related healthy city and healthy urban planning initiatives should consider the importance of healthy food environments and the detrimental impact of food deserts in their planning [80].

### Food advertising and marketing

Children’s food requests were impacted by marketing and advertising and this consequently impacted parents’ food purchases. Knowledge of food brands is a precursor to food requests and previous research shows high levels of visual recognition of brands in young children. Children are also more likely to remember unhealthy brands rather than similarly advertised healthy brands [81]. Marketing of foods high in fat, salt and sugar is often targeted at specific ethnic and lower socio-economic groups [82, 83]. It would be prudent to ensure that monitoring of food marketing towards children and related legislation should consider health inequities [15].

### Time constraints

Despite the desire for a balanced diet and the appreciation of home-made food, lack of time was a perceived barrier to healthy eating for parents. Parents’ busy schedule was a common thread across studies which contributed to consumption of convenience foods, such as pre-cooked meals and fast-food. This is reflective in survey data of parents [12, 13] and in a systematic qualitative review of dietary patterns of infants and young children where lack of time to cook was an important barrier to recommended complementary feeding practices as perceived by parents in low-income settings [84]. Additionally, as highlighted by Beck and colleagues, time constraints, that are mainly experienced by women working in low-wage jobs due to lack of flexibility in work schedules, are a significant risk factor for food poverty (i.e. limited access to enough and nutritious food for an active healthy life) [85, 86]. Therefore, it is imperative that future research aiming to improve food decisions among disadvantaged families consider lack of time as a barrier and seek ways to accommodate this in interventions, including flexibility with timing of interventions to account for parents’ schedules.

### Financial Constraints

Economic difficulties had a negative impact on family food security and dietary quality. Low-income families are less likely to make food purchasing choices in line with dietary guideline recommendations compared to those with higher household income [87]. Financial constraints experienced by disadvantaged families impeded their ability to purchase enough food of high nutritional value. Fruit and vegetables were considered expensive, which aligns with results from a previous thematic synthesis with a focus on parents of pre-school children [88]. The availability of fruit and vegetables within the home has been shown to correlate positively with their consumption and preference in school-age children, indicating that this is an important dictator of food choice [4, 89, 90].

Ultimately, the cost of food and parents’ economic capacity were discussed as important drivers of food choice, more so than the need to provide their family with a balanced and nutritious diet. Another qualitative evidence synthesis found similar results in low-income samples similar to those included in the present review, where practicalities related to money often spoiled good intentions in relation to health [88]. Observational studies have repeatedly demonstrated that low-income families are more sensitive to price than those with higher incomes and thereby more likely to choose less healthy foods [91].

### Food programmes

Food assistance programmes and government food schemes were discussed as a substantial help with food bills and offered children a diet of better quality, as has been previously shown in a longitudinal analysis in the US [92]. Even though there is great heterogeneity between food assistance services across high-income countries around the world, food provision (or vouchers for healthy foods) can significantly contribute to the diet of economically disadvantaged families. This has been previously shown in longitudinal analyses of US [92] and UK data, where government food assistance programmes (WIC and Healthy Start respectively) have significantly increased family intake of fruit and vegetables and dietary diversity [88]. This evidence suggests that there is a need for consistent policies that ensure that low-income families have access to fresh food, and enforcement of these policies must be monitored in order to improve the food environment around children [93]. While, food assistance programmes, such as food stamps and food subsidies, are useful in the short-term in helping parents to achieve nutritional sufficiency and quality for their children, they are not a long-term solution and are considered a symptom of government inaction to tackle the underlying causes of food poverty [94].

It is evident from this review that parents perceive specific factors within and outside the home to influence family food choices. While parents may have control over how and where they feed their children, the fundamental issue for low-income families is poverty, food prices and access to food outlets. While future nutrition interventions can support families to understand more about healthy eating, poverty and high food prices are the root causes of dietary inequalities and result in less choice from a restricted range of foods. Without addressing these structural issues, sustainable improvements to dietary habits of low-income families are unlikely.

## Limitations

The present synthesis of qualitative evidence on parental experiences and perspectives is essential to gain a deeper understanding of the context in which low-income families make food decisions. However, this paper is not void of limitations.

The review captured the views of low-income families, the majority of which live in the US. Findings may not be as relevant to low-income families from outside the US. Additionally, the sample of the selected studies consisted mainly of mothers. Indicatively, in the studies that included mothers and fathers, the proportion of fathers ranged from 5 to 14%. From the studies collecting data through focus groups, only one had focus groups with fathers exclusively [41]. Including paternal perspectives to a larger extent would offer a more well-rounded illustration of family experiences and future studies should focus on adopting approaches to targeted fathers as participants.

Studies retrieved using our search strategy were limited by the coverage of the search terms used. It should also be noted that authors set out to conduct a quality appraisal of all included studies in order to identify common misreported areas within the included literature. No papers were excluded from the final analysis on the basis of quality appraisal. Even though this contributed to a more well-rounded synthesis, it is possible that the inclusion of studies of poorer quality has compromised the strength of this review’s findings.

## Conclusions

Despite the limitations, this paper offers an in-depth synthesis of perceived home and community food environmental factors that drive food decisions among low-income parents with children. The perceived strong influence of food marketing and advertising on children was highlighted in addition to responding to children’s preferences and requests and the negative effect of busy schedules and limited time. Community food environments, often populated by fast-food outlets and small shops that stocked convenience foods, were limited in their ability to provide a range of healthy foods and were characterised by the high availability of foods high in sugar, salt and fat. Food cost was perceived by parents to be the primary influencer of food decisions; and economic disadvantage, the high cost of fresh food, along with limited environmental food availability had a negative effect on families’ dietary intake and quality. To our knowledge, this is the first qualitative synthesis of parental perspectives of the food environment and their influence on food decisions among low-income families. The high prevalence of racial and ethnic minority participants in this review also points to structural inequality. Findings have implications for public health, as they provide researchers and policy makers with important considerations in relation to the development of effective strategies to improve the dietary habits of disadvantaged families. Most importantly, policy and government responses to (food) poverty, food prices and access to food outlets are needed. The social determinants of health need greater recognition and attention from policy makers to enable parents to provide healthy food for their families.

## Data Availability

Any data extracted during the rapid review process can be provided for review. The extracted data analysed during the current study are available from the corresponding author on request.
